# The influence of entrepreneur’s innovation and entrepreneurship on modern art teaching model

**DOI:** 10.3389/fpsyg.2022.978821

**Published:** 2022-09-14

**Authors:** Xuan Zhang, Lin Wang

**Affiliations:** ^1^Art College, Southeast University, Nanjing, China; ^2^Faculty of Creative Industries, Malaysia City University, Petaling Jaya, Malaysia; ^3^Department of Cultural Management, Sichuan Vocational College of Art, Chengdu, China

**Keywords:** innovation and entrepreneurship, modern art education, art education model, teaching model research, students’ thinking

## Abstract

It is necessary to explore the significance of innovation and entrepreneurship to Chinese art education. The organization and operation mechanism of innovation and entrepreneurship education (IEE) is studied according to the current situation of IEE in Chinese art colleges and universities. The IEE system of art colleges and universities is optimized, and a new teaching model of IEE with the characteristics is explored. In addition, the research methods are theoretical analysis, comparative analysis, and empirical analysis. The objects are students from some domestic art academies. The influence of innovation and entrepreneurship on the modern art teaching model is explored. It mainly investigates the awareness and needs of college students’ innovation and entrepreneurship, the professional knowledge of students participating in related activities, the transformation of achievements, and the system and management of IEE. Based on this, a new teaching model of innovation and entrepreneurship is proposed. The six dimensions of creativity, initiative, interest, ideation, independence, and concentration of students in this model are evaluated. The results show that the spirit of innovation and entrepreneurship has a significant role in improving the contemporary art education model on these six dimensions. It is found that this spirit is vital for the development of contemporary art education. In art education, the application of entrepreneurship improves students’ thinking and practical ability. And this spirit is an important part of the construction of contemporary art education.

## Introduction

In the International Symposium on Education in the 21st Century, United Nations Educational, Scientific and Cultural Organization (UNESCO) believed that in addition to the traditional academic and vocational education, young people in the 21st century should also obtain “the ticket to the third education, that is, Creative Entrepreneurship” (Nia and Rahbarianyazd, 2020). The introduction of the concept of “the ticket to the third education” reformed higher education and promoted the new exploration of talent training methods in higher education (Hu and Yang, 2022). In 1995, the “Policy Document on the Reform and Development of Higher Education” published by UNESCO stated that the model of “education = work” no longer existed. College graduates became not only job candidates but also entrepreneurs and job creators. This marked a profound change in the educational methods of higher education in China, and it would be a brand-new educational concept, talent concept, and teaching concept. As the first country to carry out innovation and entrepreneurship education (IEE), the United States had gone through more than 50 years of development. Its educational system and educational concept had been formed (Liu et al., 2019).

In art management, IEE has become an important link. Especially in recent years, the idea of “innovation” has been widely recognized with the guidance of the national strategy of “innovation-driven development.” The “innovation and entrepreneurship” education of college students has also achieved unprecedented development. In recent years, many Chinese universities have set up “innovation and entrepreneurship colleges” to integrate “innovation and entrepreneurship” education into the whole process of “talent training” (Wu et al., 2020). The IEE is a new type of teaching thought and teaching method with quality education as the core. The IEE of college students emphasizes the unity of knowledge and action and pays attention to the comprehensive development of students, which conforms to the development requirements of the times. Various countries have regarded entrepreneurship education as an effective means to promote economic development and improve the quality of education with the development of the global economy and the fierce international competition. The professional characteristics of art schools and entrepreneurial education can be well combined. An entrepreneurial education model with artistic characteristics is proposed combined with the rules of artistic talent training. IEE is an innovative educational development model. After years of practice and exploration, it has achieved excellent results in many aspects. Meanwhile, it has been recognized by more and more colleges and universities. However, there are still some colleges and universities that are lagging behind in development, do not have a good understanding of the innovative talent training model, and lack a deep understanding of the development and innovation of IEE. Therefore, it actively explores the construction of an innovation and entrepreneurship for the construction of art professional talent training mode, which will help to promote the effective development of IEE’s integration into art professional education. At the same time, it also plays a positive role in promoting the education and teaching reform of the art major, which is conducive to the cultivation of high-quality talents in art colleges and universities, promotes the all-round development of students, and creates more favorable conditions for students’ future career development. And it also cultivates more innovative professionals for China.

It reports the educational thought of “creative entrepreneurship” from the aspects of talent training in art colleges and universities. The IEE of art colleges should adapt to the development of vocational education. Art colleges should realize the comprehensive development of students by making full use of their professional advantages. The concept of entrepreneurship education will be innovated based on the talent training of art colleges with art as the driving force. The innovations are: (1) the development direction of IEE in art colleges is determined, and theoretical research is expanded and supplemented. (2) A creative and entrepreneurial education model with artistic characteristics is established using multidisciplinary research methods such as art, management, education, and sociology. (3) The idea of the combined development of IEE in art colleges is proposed.

## Literature review

Innovation and entrepreneurship education courses were carried out relatively early in foreign countries. In 1947, Harvard Business School opened a “New Venture Management” for MBA students, which was considered by innovation and entrepreneurship researchers as the first course in IEE in American universities, and it was also the first appearance of IEE in a university. The course has always been the focus of IEE research in domestic and overseas. To sum up, the research mainly includes the following contents. Regarding the research on the connotation of IEE, Saji B S, Nair A R extracted that the basic connotation of IEE was a new educational concept and model with the core of cultivating students’ innovative and entrepreneurial consciousness, spirit, and qualities. They aimed to comprehensively reform traditional education and teaching and effectively cultivate innovative and entrepreneurial talents (Saji and Nair, 2018). Wei X et al. proposed to conduct an in-depth analysis of the two concepts of “innovation in a broad sense” and “entrepreneurship in a broad sense.” In a broad sense, innovation has become the hub of entrepreneurship, reflecting the intrinsic connection and essential interoperability between innovation education and entrepreneurship education, which is the theoretical and practical basis of IEE (Wei et al., 2019). Liguori E believed that IEE is a new path of employment education, and its essence is to cultivate students’ innovative spirit and entrepreneurial awareness, and it is the expansion of employment education. Scholars have different interpretations of the concept of IEE, but they all emphasize the cultivation of innovative consciousness and the importance of IEE in promoting individual development and teaching reform (Liguori and Winkler, 2020).

In the IEE system, in 1947, Harvard Business School took the lead in opening an entrepreneurship course in MBA teaching “New Venture Management,” which opened the door to entrepreneurship education in the world. Entrepreneurship education at Stanford University officially started in 1949, and other universities also offered related courses. In the IEE system, American scholars Yiyang L and Shu B pointed out that “IEE focuses on cultivating students’ entrepreneurial spirit. The education curriculum system covers both inside and outside the classroom. The launch of a variety of extracurricular projects, activities, and auxiliary courses is to more effectively achieve the effect of experiential entrepreneurship education” (Yiyang and Shu, 2019).

In terms of IEE teaching methods, the main teaching methods of foreign IEE include classroom discussion, formulation of business plans, expert lectures, case analysis, and competitions. In terms of entrepreneurship education curriculum, Afeli S A and Adunlin G proposed that the curriculum should be organized first “taking into account the psychological needs of students. Students with entrepreneurial thinking are independent individuals who do not like to be restricted, and they have the ability to think creatively, especially under conditions of ambiguity and uncertainty. They need to develop better communication skills and better instruct others on how their actions are perceived. Therefore, entrepreneurship courses should not be rigidly structured, but instead present students with real-world entrepreneurial issues in an ambiguous and risky environment, thereby encouraging students to think independently” (Afeli and Adunlin, 2022).

In the aspects of the evaluation system of IEE, Torres pointed out in the “Critical Evaluation of Two Entrepreneurship Education Models,” after comparing the two models with the purpose of integration, he made an in-depth qualitative analysis of the structure of each model, and evaluated the contributions and limitations of each model, and then proposed an integrated form of the model. In “Exploring Non-Traditional Approaches of High-Level Entrepreneurship Education,” Lao expanded the stakeholders of entrepreneurship education program evaluation to policy-makers, government authorities, regional developers, etc.

For the values and goals of the IEE curriculum, this type of research is mainly concentrated in the early research of foreign scholars. For example, since the 1960s, foreign scholars have carried out intense discussions on “whether innovation and entrepreneurship can be taught,” and finally reached a consensus in the academic community that “innovation and entrepreneurship can be taught.” This realization also drives the IEE from value to target research. Furthermore, Varano M et al. ranked the importance of IEE curriculum objectives and believed that “improving students’ cognition and understanding of the process of enterprise creation and management” and “improving students’ understanding of entrepreneurship as a career choice” are vital objectives of the curriculum. The research conclusion of Jeffery A. Timmons, a leader in entrepreneurship education in the United States, pointed out that the purpose of entrepreneurship curriculum design is to cultivate students’ entrepreneurial ability (Varano et al., 2019). Ghafar A argued that the entrepreneurship education ecosystem is based on universities, including individuals, organizations, external entrepreneurial culture, resources, and other elements, and its core is entrepreneurial activities and courses (Ghafar, 2020).

Regarding the empirical analysis of innovation and entrepreneurship in the art teaching model, China’s IEE started late, and there are few IEE literature on art. According to the statistics of CNKI in this paper, from January 2019 to January 2022, a keyword path search was conducted with “IEE” as the search term, and 32,598 articles were found; the keyword search was carried out with “IEE of Art” as the search term, and 200 articles were found. According to the calculation, the proportion of articles published in core journals by journals about “IEE” is 13.2%, which is relatively small; the number of journals about “IEE of Art” is relatively small, and the number is only 11, accounting for only 5.5%. Therefore, it can be seen that the research on IEE in China is still in its infancy. Among them, Zhanren Wang, in his book “History of IEE in China,” from the standpoint of the history of IEE development and rooted in the reality of China’s IEE development, it comprehensively sorts out and analyzes the historical origin, local creation, educational practice, development and expectations of China’s IEE. In addition, his book “Introduction to IEE with a Broad Spectrum” explored the realistic path of IEE in terms of t in terms of IEE curriculum system, teaching staff, training methods, and evaluation system (Almahry et al., 2018). Zhichao Liu et al. considered that under the new situation, cultivating more art and design professionals with innovative spirit and ability is the key factor for the employment of college students. The innovation and practice of cultivating professional talents also require that the art design major should regard practical teaching as the key content. Specifically, it is necessary to attach significance to the cultivation of students’ innovation and entrepreneurship ability. An important prerequisite for further improving the level of practical teaching is the construction of a practical teaching system for innovation and entrepreneurship (Zhichao et al., 2020).

Innovation and entrepreneurship education is a process of interaction between teachers and students. This process can be summarized as “co-creation between teachers and students.” To correctly understand the feasibility of co-creation in IEE, it needs to accurately understand the profound connotation of IEE. It is a new teaching concept and model that aims at cultivating talents with entrepreneurial awareness and pioneering spirit. Its essence is not only to solve employment problems, but also to cultivate students’ entrepreneurial awareness and practical ability. In 2010, the Chinese Ministry of Education renamed this education as “IEE,” indicating that a consensus has been reached on the twin nature of IEE. It inherently stipulates the application attributes of innovation, which is entrepreneurial-oriented innovation, focusing on innovation in practical application, and emphasizing the marketization and commercialization of innovation results. The word “innovation” is added in front of “entrepreneurship,” and its essence is that innovation comprehensively dominates the direction of entrepreneurship, that is, innovative entrepreneurship, which improves the level of entrepreneurship. As the “Father of Entrepreneurship Education” Timmons pointed out: “If entrepreneurship is compared to the engine of the American economy, then innovation is its cylinder, which drives the birth of important new inventions and new technologies.” Therefore, the core connotation of IEE should be aimed at implementing a modern higher education model oriented toward cultivating top-notch innovative and entrepreneurial talents, guiding teachers and students in schools to continuously update and sublime educational concepts, and leading education and teaching reform with innovation and entrepreneurship. It closely integrates the four functions of talent training, scientific research, social service and cultural inheritance in colleges and universities, realizes the transformation from simple knowledge transfer to more emphasis on ability and quality training, and strengthens the cultivation of students’ innovative entrepreneurial spirit and ability. To effectively improve the overall quality of talents, IEE is inseparable from the deep participation of teachers in the whole process. Continuous improvement of teachers’ innovation and entrepreneurship ability is the premise of implementing high-level IEE. It is a process of mutual promotion and common progress between teachers and students. Regarding the influence of traditional culture on IEE, there are two representative viewpoints in the academic circles. On the one hand, from the perspective of traditional culture’s “robust and promising” spirit, it is believed that the Chinese nation is an innovative nation, and Chinese culture has always attached great importance to innovation and creation. Traditional culture contains rich creativity. On the other hand, it is based on the “inferiority” of traditional culture, which believes that traditional culture lacks genes that encourage innovation, resulting in a lack of creativity. Although there are cognitive confrontations between these two viewpoints, they both recognize the positive and negative aspects of traditional Chinese culture. Due to the different perspectives, the same cultural characteristics have diametrically opposite effects on the cultivation of innovation and creativity.

Most foreign scholars think that the idea of innovation and entrepreneurship can benefit graduates of all majors for life. Not only is it backed by high-level EU policy documents, but research shows that the development of the IEE does have a positive impact on students’ careers and social progress. Professor Jeffry Timmons proposed that entrepreneurship education is to set an “entrepreneurship genetic code” for future people, cultivate students’ innovative spirit and entrepreneurial ability, and be oriented to improve students’ entrepreneurial cognitive ability. To sum up, the IEE of foreign vocational colleges not only emphasizes cultivating students’ innovative spirit, but also pays more attention to cultivating their entrepreneurial ability. American scholars Solom and Sid argue that professional education is the systematic and socialized education of learners’ professional ideas. It can improve the academic ability of students to accurately judge the specific things of specialization, and finally be able to take appropriate measures independently. It can be seen that research on the impact of innovation and entrepreneurship on education models is not uncommon. On this basis, the influence of innovation and entrepreneurship on the teaching model of art students is studied to make new discoveries and breakthroughs.

## Materials and methods

### Empirical analysis of innovation and entrepreneurship in the art teaching model

#### The essence of innovation and entrepreneurship education in art colleges

Some scholars have discussed entrepreneurship education with the continuous deepening of it in academia. They believe that innovation is an academic field, and entrepreneurship is an applied field. If the two are combined, there is no clear direction. The “broad” and “narrow” connotations of IEE should be correctly grasped (Zhou, 2021). In a broad sense, “IEE “is an educational practice to create a new and great cause. In a narrow sense, it refers to a teaching practice activity that creates a new type of occupational employment in cultivating students’ independent employment, flexible employment, and self-employment. IEE is an inevitable requirement of quality education and a new understanding of quality education. It is a specific, real, targeted, and implementable new designation (Zhang, 2020). The IEE is a brand-new educational thought and educational method in today’s higher education reform. IEE in art colleges presents the following characteristics compared with IEE in comprehensive universities (Vandor, 2021).

**FIGURE 1:**
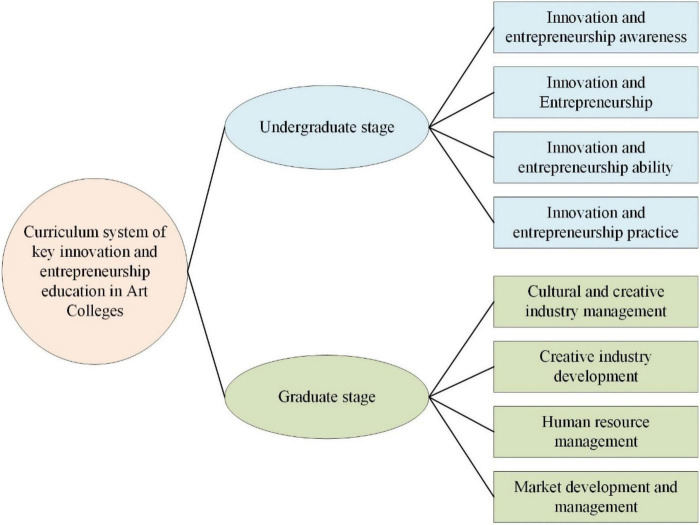
The curriculum system of key IEE in art colleges.

The first is to focus on cultivating innovative consciousness and entrepreneurial spirit, so college students can transform from passive applicants to active creators. It is emphasized that innovation and the pursuit of beauty and art are combined with vocational education (Higgins Desbiolles, 2018). The second is to focus on combining IEE with professional education. The IEE is integrated into specific professional courses when courses in innovative thinking, entrepreneurial management, corporate finance, corporate strategy, project, and operation and management are offered (Wu et al., 2021). The third is to cultivate students’ emotional experience. Students experience the entire process of entrepreneurship by holding various innovation and entrepreneurship competitions and training camps (Feng and Feng, 2021). The fourth is the result of the rapid transformation of academic research and artistic creation into science and technology. The IEE of the art colleges emphasizes the combination of production, learning, and research. The service function of the arts is used to transform students and their research results into scientific productivity, providing strong support for local economic development (Yefimenko and Yakymchuk, 2020). Figure 1 shows the curriculum system of key IEE in art colleges.

#### The purpose of innovation and entrepreneurship education in art colleges

**FIGURE 2:**
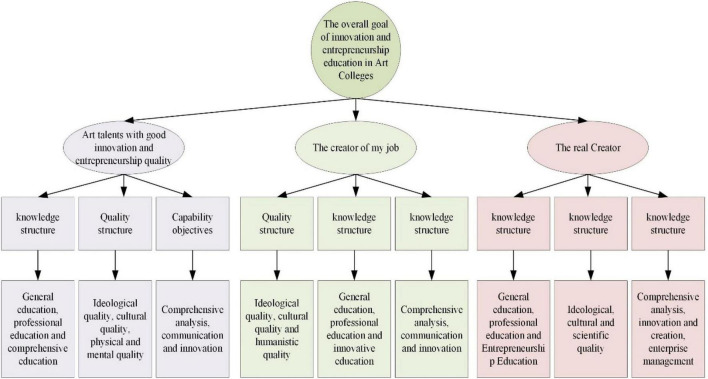
Hierarchical goals of IEE in art colleges.

The essence of IEE is to cultivate the comprehensive innovation and entrepreneurship ability of college students. Therefore, life experience plays an important role in their growth. Much knowledge needs to be gained from experience (Karhio, 2021). Humanists believe that “education begins with experience.” Experience is very vital. Likewise, much knowledge cannot be taught and instilled but must be experienced by students (Xu et al., 2021). Knowledge cannot bring creativity, and true innovation should start with experience. Creativity is achieved through a deep understanding of art. Creation is the foundation of art colleges, and creative education is the vocation of art colleges (Wadoski, 2020). In the final analysis, IEE in art colleges must return to “people.” Entrepreneurship education should realize the economic interests of “people-oriented,” “highlight” the “subject” of students, and show their “dignity” and “value” to serve the “harmonious development” of “high rationality” and “high emotion” (Kurpayanidi, 2021). The IEE in art colleges should comprehensively improve the innovation and entrepreneurship quality of college students, stimulate their subject spirit and potential, and create a good ecological environment. The ultimate goal is to cultivate innovative and entrepreneurial compound talents with aesthetic awareness and noble sentiments (Zheng and Ke, 2020). The hierarchical goals of IEE in art colleges are displayed in Figure 2.

#### Approaches to innovation and entrepreneurship education in art colleges

**FIGURE 3:**
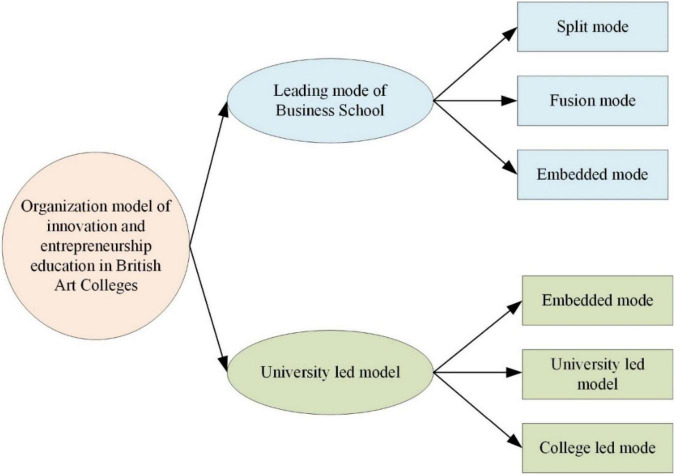
The IEE model of art colleges.

The IEE of art colleges establishes an ideal framework of “who I am” for college students. It matches and adapts to the characteristics of college students in terms of teaching content, methods, and process (Dai, 2020). From the perspective of teaching content, IEE in art colleges focuses on comprehensiveness (Ye and Chen, 2021). It is not a simple combination of management disciplines such as marketing, human resources, finance, and quality control but a complete system built around the entire life cycle of a company. Discrete functional courses are combined to help readers cope with the seemingly disorganized and unpredictable entrepreneurial process based on the entrepreneurial process model (Yu and Wang, 2021). Experiential education is a people-oriented teaching concept. It guarantees students the realization of their learning rights through hands-on experience. Subjectivity is maximized (Karhio, 2021). The methods suitable for instillation are acceptance and memory, while those suitable for experience are exploration, discovery, creation, and transcendence. It is a positive, non-compliant, non-conservative attitude that is creative and transcendent (Liu and Chen, 2018). Figure 3 suggests the IEE model of art colleges.

#### Methods of improving students’ awareness of innovation and entrepreneurship in art colleges

##### Innovation and entrepreneurship education integrates the new concept of professional education

The high integration of IEE into professional education should be mainly reflected in the fit of educational concepts. The specific manifestation is a new type of education and teaching concept, that is, the core education and teaching concept of “innovation + entrepreneurship + professionalism.” First, the traditional cognitive concept of professional education has been actively changed, and the differences and similarities in the educational concept of IEE and professional education have been recognized. The similarities between the two are the education models for talent cultivation. The differences are mainly reflected in the following aspects (Akyavuz and Asc, 2021). IEE emphasizes cultivating learners’ awareness of innovation and entrepreneurship, cultivating students as innovative and entrepreneurial talents. Professional education emphasizes the mastery of learners’ professional skills and abilities, and then cultivates them into professional talents. Therefore, instead of just emphasizing a single talent training program, the two concepts of talent training and education should be effectively combined. Second, an efficient talent training plan should be set up to guide the government, enterprises and schools to actively participate in it, and establish a variety of talent training goals. The first draft of the plan shall be drawn up by the colleges and universities according to the requirements and plans of the talent training plan, and the relevant personnel of the government and enterprises shall make suggestions. Then, a talent training plan that is recognized by the “government, school and enterprise” is formed. Third, the talent training goals finally formed in its plan should be diverse, taking into account the individual needs of students, so as to meet the local characteristics of each college and the differences of students (Zhong et al., 2020).

##### Innovation and entrepreneurship education is integrated into the curriculum system of professional education

Innovation and entrepreneurship education is organically integrated into professional education. Its curriculum system should be gradually improved. Hence, a diversified talent training plan with “IEE organically integrated into professional education” should be formulated. The goal is to cultivate high-quality talents who not only possess the ability of innovation and entrepreneurship, but also be able to apply professional theoretical knowledge proficiently, and accelerate the organic integration of IEE into professional education. At the same time, a talent training program based on improving the awareness of innovation is formulated. The curriculum system in which the theory of “offline training supplemented with online guidance, extracurricular practice combined with in-class teaching, and optional courses combined with required courses” is increasingly being applied to practice. With the improvement of the IEE system, the comprehensive quality of students has been effectively improved. To organically integrate IEE teaching into professional education and teaching, professional education should make full use of IEE resources inside and outside the school according to its own characteristics, so that the implementation of IEE teaching courses can play a greater role and accelerate the organic integration of the two.

##### Innovation and entrepreneurship education is integrated into the teaching staff of professional education

With the reform of IEE in secondary vocational colleges, at present, these colleges need to build a “full-time and part-time, on-campus and off-campus” teachers who not only possess certain professional theoretical knowledge and ability, but also have rich practical skills. In the college, each major selects entrepreneurs with certain professional theoretical ability, or has successful and rich entrepreneurial experience and professional teachers to jointly teach courses of IEE. Meanwhile, it recruits outstanding talents with IEE experience or alumni entrepreneurs in the field to strengthen its faculty. At this stage, secondary vocational colleges should attract a number of entrepreneurial mentors to provide guidance, strengthen students’ awareness of innovation and entrepreneurship, and enhance their willingness to practice. Students’ innovation and entrepreneurship are created with certain objective conditions, which strongly support their entrepreneurship.

#### Methods to improve art students’ understanding of innovation and entrepreneurship

The first is to inspire the innovation and entrepreneurship awareness of art students. Subjective innovation consciousness and entrepreneurial spirit are the fundamental driving forces for the formation and promotion of college students’ entrepreneurial behavior. Only with the foundation of innovation and entrepreneurial consciousness will entrepreneurial behavior gradually emerge. Therefore, improving college students’ innovative and entrepreneurial consciousness is crucial to their understanding of innovation and entrepreneurship. For colleges and universities, they should first guide college students to generate and enhance their own innovative consciousness and entrepreneurial spirit, so that they have the awareness of using knowledge and wisdom to create a career that can give full play to their personal strengths. Second, they should be realized that the employment situation is severe. At present, to better adapt to the era and realize self-value and development, it is necessary to strengthen one’s own innovation consciousness, and entrepreneurial spirit, and choose the correct career development route.

The second is to strengthen the construction of teachers and build innovation and entrepreneurship courses. The professionalism and conscious guidance of teachers play a vital role in improving the practical ability of innovation and entrepreneurship of art students. The quality level of teachers is an extremely important factor for improving talents with innovative and entrepreneurial abilities. Teachers must keep abreast of the cutting-edge knowledge of the subject and impart it to students as soon as possible, to achieve the goal of talent training to a certain extent. At the same time, the teachers’ way of thinking and the details of how to deal with problems will also indirectly affect the students, helping them to have a deeper understanding of the spirit of innovation and entrepreneurship.

The third is to create a good platform for improving students’ practical ability of innovation and entrepreneurship. To cultivate the innovation and entrepreneurship ability of art students, it is very important to build a practical platform. Therefore, art teachers should adhere to the combination of classroom and extracurricular, and combine social practice with art practice. It can give full play to the role of art student associations, carry out rich extracurricular activities, encourage students to organize innovation and entrepreneurship scientific research activities, to broaden students’ horizons and mobilize their enthusiasm, and gradually form a good cultural atmosphere for innovation and entrepreneurship. Practice is the most powerful way to deepen understanding.

### Empirical analysis of innovation and entrepreneurship education in Chinese art colleges

#### Subjects of the questionnaire survey

It studies from innovation and entrepreneurship awareness, ability, and management mechanism of art college students. The first is to discuss the interests, motivations, needs, and influencing factors of the current situation and problems of innovation and entrepreneurship of students in art colleges. The second is the current situation of the management personnel of art colleges in the process of carrying out IEE. Eight representative colleges are selected to investigate the current situation of IEE from practical needs. A detailed analysis of influencing factors is carried out combined with the results of the survey. A total of 800 questionnaires are distributed, and each university distributes a total of 100 questionnaires, of which 311 are male, and 457 are female.

#### Formulation of the questionnaire survey

Total of 768 questionnaires were effectively recovered, and the effective rate was 96%. A total of 9 sets of questionnaires were distributed and effectively recovered, with an effective rate of 100%. The data of this questionnaire survey were processed by Excel statistics, and the data results were analyzed. The colleges and universities involved in this survey include Central Academy of Fine Arts, China Academy of Art, Xi’an Academy of Fine Arts, Luxun Academy of Fine Arts, Hubei Institute of Fine Arts, Tianjin Academy of Fine Arts, Guangzhou Academy of Fine Arts, and Sichuan Fine Arts Institute. There are 311 males and 457 females. The survey respondents include students from freshman to senior year, including 236 urban students and 532 rural students; 83 students whose parents have started a business, and 685 whose parents have never started a business; There are 695 students who have offered entrepreneurship courses, 60 students who have not; there are 13 students who are not clear about whether to offer courses; 98 students have entrepreneurial experience themselves, and 670 students have not.

It mainly discusses the factors that affect college students’ entrepreneurship, the setting of entrepreneurial courses, the influence of entrepreneurial services, and the entrepreneurial service system. Some research results have been achieved. Moreover, the influencing factors of the entrepreneur’s personality characteristics are analyzed, and six main factors that affect the entrepreneur’s personality characteristics are summarized. These factors mainly include creativity, initiative, interest, ideation, independence and concentration of art students. These six factors are independent but related to each other. For example, art students’ interest in majors will affect their initiative and concentration in learning, and students’ ideation is closely related to their creativity. It can be found that these six factors are inherently related, and have a certain impact on students’ innovation and entrepreneurship from different aspects. It is of great significance and role to analyze the impact of these factors on the teaching model of art students.

#### A survey on the field of innovation and restrictions on innovation and entrepreneurship of art students

The questionnaire is designed to investigate the relevant content of the current art teaching innovation. It aims to understand what aspects of art students need to innovate, and to provide ideas and a basis for follow-up research. Meanwhile, 100 art students and 100 graduate job seekers in this major in the past 3 years were selected to investigate the factors that limit their innovation and entrepreneurship, hoping to obtain the main reasons that affect the innovation and entrepreneurship of art students. The results of men and women in the above two surveys are compared to verify whether there are any differences in their innovative spirit.

## Results and discussion

### The innovative principle of the teaching model

#### The principle of adaptability

At present, teachers mainly teach courses according to professional training plans and teaching plans in the teaching of vocational education for art students in China. Therefore, organically integrating IEE into professional education should not only pay attention to the systematicness and rationality of professional talent training programs but also consider its practicality and comprehensiveness (Aziuk, 2021). Art colleges should organically integrate IEE into the professional talent training programs of various disciplines in terms of teaching content, teaching design, credit setting, and practical training courses. The advantages of IEE are fully used in the professional teaching link to further improve the comprehensive quality and practical ability of students and effectively promote the efficiency of talent training (Gou and Sun, 2021). In the future, Chinese art education should take IEE as quality education. Innovation and entrepreneurship generally exist in various organizational activities. Using the spirit of innovation and entrepreneurship to carry out work is the prerequisite for progress. Innovation and entrepreneurship should cultivate and strengthen students’ innovative thinking and entrepreneurial ability, identify and grasp various entrepreneurial opportunities, and learn to creatively integrate various resources. This is undoubtedly a new type of quality education. Therefore, in the process of reforming the art teaching model, the IEE model should be adapted and constantly broken through. The impact of innovative courses on entrepreneurs, and the results of students’ entrepreneurial awareness and needs are shown in Tables 1–3.

**TABLE 1:** The opening of innovation and entrepreneurship courses in eight art colleges and their impact on entrepreneurs.

Innovation and entrepreneurship courses	Course offerings (with or without offering)	Impact on entrepreneurship (with or without impact)
Management	No	Yes
Economics	No	Yes
Legal	No	Yes
Entrepreneurship	Yes	Yes
Career guidance	Yes	Yes
Psychological	No	Yes
Other courses related to innovation and entrepreneurship	Yes	Yes

**TABLE 2:** Statistical table of innovation and entrepreneurship awareness among students from the eight art colleges.

Investigate subject	Number of people	Number of questionnaires	Proportion (%)
Understand IEE	175	768	22.79%
Interested in innovative and entrepreneurial activities	185	768	24.12%
Innovation and entrepreneurship activities are necessary and rewarding	125	768	16.38%
Learn about relevant innovation and entrepreneurship policies	76	768	9.9%
Entrepreneurship is far away	468	768	60.94%
Have innovative ideas	320	768	41.67%
Number of students who have started a business	45	768	5.85%
Entrepreneurship drives employment	20	768	2.60%

**TABLE 3:** Statistics of demand for innovation and entrepreneurship of college students from the eight art colleges.

Investigate subject	Number of people	Number of questionnaires	Proportion (%)
Access to sufficient funds	700	768	91.46%
Perfect knowledge structure	752	768	97.92%
Family support	638	768	83.07%
Practical training and experience	671	768	87.37%
Innovation and entrepreneurship guidance and services	586	768	76.30%
Support policies	677	768	88.15%

#### The principle of service

Innovation and entrepreneurship education should serve social practice. By imparting the theoretical knowledge of innovation and entrepreneurship, IEE enables students to understand the inherent laws and key issues of entrepreneurship, and possible risks, and helps students plan their careers scientifically and reasonably. Those cases of entrepreneurial failure are not because the students’ entrepreneurial projects are not good, nor because there is no market, but because the students do not know how to transform innovative technologies into social value. Many innovative technologies cannot be transformed into real productivity. IEE is to teach knowledge and skills to help students achieve personal value while enhancing social benefits.

#### The principle of step by step

The organic combination of IEE and vocational education is the internal demand of vocational colleges to improve the quality of talent training with the deepening of the reform of vocational education. This is also an important measure to promote the reform of personnel training methods in vocational colleges under the concept of innovative development. Any form of education and teaching talent training program must be based on the law of human development (Amirkhanov et al., 2020). Therefore, organically combining IEE with vocational education should be a process of boldness, innovation, and exploration. Vocational colleges should follow the principle of step-by-step, change concepts, and continuously innovate to satisfy the real needs of higher vocational talent training reform and stimulate students’ entrepreneurial enthusiasm and creativity (Halvorsen and Chen, 2020). According to the above survey, there are many types of courses that affect the innovation and entrepreneurship of art students. In the course of learning these courses, students gradually accumulate relevant knowledge, and have more new experiences and feelings from various aspects such as understanding and cognition, gradually cultivating students’ creative thinking and ability, thereby playing a role in promoting the IEE of art students.

### Analysis of survey results

The awareness of Innovation and entrepreneurship refers to a strong will, interest, and attitude that college students have in their entrepreneurial activities. It is the source of creativity in human thought and consciousness and the inner driving force for creative and entrepreneurial activities. The results indicate that there are some problems in the awareness of innovation and entrepreneurship among students in the eight art colleges.

The survey results reveal that 22.79% of the students have a clear understanding of the concept of entrepreneurship, and 24.12% of the students are interested in entrepreneurial activities. 16.38% of the students believe that it is necessary and beneficial to participate in innovation and entrepreneurship activities. Only 9.9% of the students understand entrepreneurship-related policies, and 50.94% of the students believe that entrepreneurship is far away from themselves, so they think that IEE is optional. In addition, among them, females account for 33.6%, and males account for 17.34%. 41.67% of the students have the idea of starting a business, 15% are female, and 26.67% are male. 5.85% of the students are real entrepreneurs, of which only 1.8% are female, and 2.60% are realized through self-employment. It is found that the students of art colleges have a bias in their understanding of entrepreneurship, but their enthusiasm for entrepreneurship is higher than that of students from other colleges, and more of them are students with an entrepreneurial spirit. The proportion of students who have started a business is also significantly higher than that of students from other schools.

The results show that among college students, 93.75% believe that the funds for entrepreneurship are insufficient. The proportion of males is 41%, and females are 52.75%. 91.46% of college students do not have the “knowledge knot,” of which 36.4% are male and 55.06% are female. 87.37% consider that “innovation and entrepreneurship” should accumulate experience by participating in social practices. 52.6% of males, 34.77% of females. 76.30% feel that they should accept the school’s accurate guidance, of which 40% are male, 36.30% are female. 88.15% think that government and school policies have played a great role in entrepreneurship. Among them, men account for 48.13%, women account for 40.02%. Therefore, there is a problem that the government, society, schools, families, and other aspects cannot meet the real needs of college students in the practice of innovation and entrepreneurship in China. The professional knowledge statistics, achievement transformation and cognitive level test results of art college students participating in innovation and entrepreneurship are shown in Tables 4–6.

**TABLE 4:** Statistics of professional knowledge of students participating in innovation and entrepreneurship activities in eight art colleges.

Investigate subject	Number of people	Number of questionnaires	Proportion (%)
Familiar with the “Challenge Cup” innovation and entrepreneurship competition	211	768	27.47%
Entries related to professional knowledge	499	768	64.97%
Entries with high technical content	335	768	44.01%
The winning work is the work of the instructor	488	768	63.54%
Access to cutting-edge knowledge	137	768	17.84%

**TABLE 5:** Statistics of the transformation of innovation and entrepreneurship achievements of the eight art colleges.

Investigate subject	Number of people	Number of questionnaires	Proportion (%)
There are innovation and entrepreneurship practice bases or parks	133	768	17.32%
Participated in innovation and entrepreneurship training and practice	127	768	16.54%
Participated in innovation and entrepreneurship activities	82	768	10.68%
Innovation and entrepreneurship projects are transferred or adopted	34	768	4.42%
Cooperate with companies to complete projects	16	768	2.08%
Art creation and output of works	462	768	60.94%

**TABLE 6:** Statistics of awareness level of IEE in eight art colleges.

Investigate subject	Number of copies	Number of questionnaires	Proportion (%)
IEE is to solve employment problems	7	8	87.5%
The IEE is a routine skill guide	6	8	75%
IEE is elite education	7	8	87.5%
IEE is an extracurricular activity and competition	6	8	75%
Integrate IEE into the whole process of talent training	4	8	50%

From Table 4, art college students’ awareness of the “Challenge Cup” entrepreneurship competition is only 27.47%. Many professional teachers participating in the competition have low levels of participation. 64.97% of the works are related to the professional skills of students, while 44.01% of the works have high skills in the preliminary selection of the innovation and entrepreneurship competition works. Mentors’ works account for 63.54%, and only 17.84% of cutting-edge knowledge could be obtained. This indicates that the technical level and overall level of the students participating in the innovation and entrepreneurship competition are low. Art college students rarely have the opportunity to participate in independent innovation in addition to participating in national and provincial innovation and entrepreneurship competitions.

In Table 5, only 17.32% of college students participate in innovation and entrepreneurship, of which 13.2% are male and 4.12% are female. Only 16.54% participate during school. Men account for 10%, and women account for 6.54%. Only 4.42% of innovation and entrepreneurship projects are transferred or accepted, and the proportion of males is 3.22%, and females are 1.10%. The proportion of cooperating with companies to complete projects is only 2.08%, of which 1.83% are male and 0.25% are female. It is concluded that the participation rate of students is low, and the conversion rate of achievements is low in the creative and entrepreneurial practice activities carried out in art colleges. From the school’s point of view, many students’ innovation and entrepreneurship are still in a state of “utilitarianism,” and they do not regard IEE as a rational understanding of long-term and stable cultivation of artistic talents. The creative entrepreneurial practice activities cannot profoundly impact the development of the local economy and society.

From the perspective of the teaching system, the professional orientation of IEE in disciplines such as business management and business administration is not clear, and the professional foundation is not perfect due to the marginalization of disciplinary status. Art colleges still face some problems in innovation and entrepreneurship. Art colleges do not have management and business management majors, and students do not have corresponding professional knowledge in marketing, financial analysis, and business management. School education is mainly technical training. The way of teaching is traditional indoctrination. School education lacks a macro grasp of the innovative spirit of college students, resulting in poor educational results. Most IEE courses are decentralized and separate from vocational education. There are problems such as lack of scientificity and systematization, low level of teachers, and insufficient financial preparation. The statistical results of the student management system in art colleges are shown in Tables 7, 8.

**TABLE 7:** Statistics of the IEE system of the eight art colleges.

Investigate subject	Number of copies	Number of questionnaires	Proportion (%)
Single course content	6	8	75%
Disconnection and separation from subject and professional education	7	8	87.5%
Insufficient teachers and low level	8	8	100%
Backward teaching facilities	7	8	87.5%
Insufficient financial support for schools	6	8	75%

**TABLE 8:** Statistics of IEE management in eight art colleges.

Investigate subject	Number of copies	Number of questionnaires	Proportion (%)
Set up a special IEE management organization	4	8	50%
Set up specialized IEE research institutions	0	8	0%
Frequent exchanges of innovation and entrepreneurship activities in various colleges and universities	2	8	25%
All majors maintain linkage with IEE	3	8	37.5%

Many art colleges do not have an independent IEE management department, and IEE is undertaken by students, youth league committees, and employment departments. Most of the IEE is in charge of the school leaders who deal with student work. School leaders in charge of teaching rarely care about IEE. There are deficiencies in the management of IEE by the educational administration department. The lack of communication between relevant departments has led to the lack of close connection between the IEE of the art colleges and the disciplines and majors, making it difficult to develop deeply.

The survey results show that IEE in art colleges is still in its infancy and has not received sufficient attention from higher-level leaders. The existing theoretical system is still at the research level and cannot be adapted to the existing teaching system of art colleges. The form and content of IEE are relatively simple, lacking a scientific and systematic theory and practice system.

According to the questionnaire survey, among the surveyed art students, the areas that need innovation in the current art teaching are expressed in Table 9. Among them, 52% of the students believe that the management of cultural and creative industries should be innovated, and 28% of the students feel that it is necessary to innovate the management of art students. In addition, the number of males and females surveyed was 1:1, and 58% of the final valid questionnaires came from males and 42% from females. 78% of male students chose the innovative practice course, and 69% of the female students chose the innovative basic course. It can be found that the innovation and optimization of cultural and creative products is an urgent task at present, and the integrated development of cultural and creative products should be strengthened.

**TABLE 9:** Statistics in the field of innovation and entrepreneurship of art students.

Course level	Course content
Foundation course	Introduction to innovation and entrepreneurship, innovation and entrepreneurship management, cultural and creative industry management
Core course	Venture capital theory, financial management, marketing management, art course management
Deepen course	Business field: business organization theory, business strategy theory, management theory; accounting field: general financial management, management accounting theory, business analysis theory; information field: business information system; economic field; microeconomics, industrial organization theory; circulation field: consumer behavior theory, modern consumer economy theory; legal field: enterprise law, economic law, etc.
Practical courses	Practical training and basic roadshows necessary for innovation and entrepreneurship and new product development

According to the survey, the main factors affecting the innovation and entrepreneurship of job seekers are life pressure and work pressure, but the main factor affecting for art students lies in themselves. The number of males and females in the survey was 1:1. Among the valid questionnaires obtained, the proportion of males was 55% and the proportion of females was 45%. The statistical results of students’ artistic innovation and entrepreneurship are shown in Table 10.

**TABLE 10:** Statistics on influencing factors of innovation and entrepreneurship among job seekers and art students.

Survey object	Influencing factors	Proportion
Job seekers who majored in arts in the past 3 years	Family and friends	12%
	Life and work stress	56%
	Interpersonal relationship	23%
	Other aspects	9%
Art students at school	Academic pressure	28%
	Own factor	45%
	Surroundings	10%
	Other aspects	17%

In addition, it also investigates and compares the number of entrepreneurship students in other disciplines and the number of entrepreneurship in art students, and finds that due to differences in majors, skills, and other aspects, there are differences in innovation and entrepreneurship between students in other disciplines and art students. On the whole, in other disciplines, especially science students have a higher percentage of entrepreneurship, which is significantly higher than that of art students.

### Analysis of the influencing factors of innovation and entrepreneurship of art students

According to the communication with art students, compared with young people who have already joined the job, the reasons that affect students’ innovation and entrepreneurship are mainly reflected in the following aspects.

#### The problem of the educational environment

The influence of the environment on people’s spiritual field is far-reaching, and the environmental problems of Chinese college students’ spiritual cultivation are insufficient. First, colleges lack an atmosphere of innovation and entrepreneurship. Nowadays, most of the IEEs in Chinese colleges and universities are to carry out innovation and entrepreneurship competitions on campus or offer some corresponding elective courses. Although some colleges give generous rewards to college students’ innovation and entrepreneurship competitions and actively encourage outstanding students with outstanding innovation and entrepreneurship capabilities, college students generally lack enthusiasm and cannot fully and extensively involve themselves in innovation and entrepreneurship. The innovation and entrepreneurship competition in some colleges is grand, but very few students actually participate in the competition. Many college students understand innovation and entrepreneurship with a spectator mentality. Such a mere formality of university innovation and entrepreneurship environment can be imagined with its effect. It is well known that holding innovation and entrepreneurship competitions is only an auxiliary means to create a spiritual cultivation environment, and cannot be the only means. Optimizing the spiritual cultivation environment of colleges and creating an atmosphere of innovation and entrepreneurship on campus is a problem that universities need to consider at this stage. Second, there is a lack of a cultivating environment for multi-party cooperation. The environment for cultivating spirit in colleges and universities should not be limited to schools. At the present stage, the environment for cultivating the innovation and entrepreneurship of Chinese college students is not unified and coordinated among society, universities, and families.

#### The problem of the education method

Recently, the research on the innovation and entrepreneurship of college students has made great progress compared with the past. However, under the constraints of the traditional concepts of universities, some problems of college students’ IEE have not been fundamentally and effectively solved. For example, the phenomenon of “contempt for education” and “formalized education” still exists, and the effectiveness of spiritual cultivation of some college students is not strong. The first is the influence of external negative factors in society. In today’s Internet age, network information is transmitted in real-time, and some unfiltered bad information has had a very bad impact on the spiritual cultivation of colleges, such as money worship and hedonism. The second is that cultivation has not gone deep into reality. Some universities do not carry out scientific and reasonable spiritual cultivation according to their own school-running characteristics, but “copy” the education model of other schools, resulting in a lack of enthusiasm for students to participate. And IEE has become a formal education, and its effectiveness can be imagined. The third is the lack of “unity of knowledge and action” in cultivation. In some universities, the theory and practice of spiritual cultivation knowledge are decoupled, resulting in college students becoming the “storage” of knowledge, lack of practical ability, and failing to “internalize the innovation and entrepreneurship of college students in their hearts and externalize them in their actions.” The IEE method for college students in universities is mostly traditional. Some public courses are set up by teachers to teach innovation and entrepreneurship knowledge, and students often listen passively. The disadvantages of this traditional cultivation method are becoming more and more obvious. This cultivation method leads to poor students’ autonomy and often fails to achieve the purpose of teaching. The core connotation of IEE should be to strengthen the cultivation of students’ innovation and entrepreneurship, consciousness, and ability, and effectively improve the quality of personnel training.

#### The problem of the education team

Teachers have an important impact on the cultivation of the innovation and entrepreneurship of college students. Looking at the relatively excellent innovation and entrepreneurship universities abroad, they have gathered a large number of excellent academic teams and a high-level teaching staff. In contrast, China’s teachers are relatively weak. A considerable number of IEE teachers in universities do not have a systematic study of innovation and entrepreneurship theory, and their recognition and support for the cultivation of innovation and entrepreneurship of college students are very low. At present, most of the innovation and entrepreneurship teachers in China’s colleges and universities are mainly other professional teachers, counselors, or managers. Due to the lack of experience of teachers in colleges, they can only teach in the classroom under limited conditions, and their guidance method in the innovation and entrepreneurship classroom is relatively simple. Even though colleges invite some outstanding entrepreneurs to give lectures in the innovation and entrepreneurship classroom to enhance the teaching effect, the actual effect is very small. Although many excellent entrepreneurs are full of innovation and entrepreneurship, objective factors such as limited classroom time, lack of systematic presentation, and lack of teaching experience by non-professional teachers have affected the actual effect.

### Strategies to promote the cultivation of innovation and entrepreneurship in art students

#### Strengthen the entrepreneurial spirit of female students

According to the above statistical results, it can be seen that the innovation and entrepreneurship spirit of female students is slightly weaker in comparison. Therefore, the cultivation of female students’ this spirit should be strengthened. Lectures on innovation and entrepreneurship can be held in schools and interested students can be invited to participate. It can also set up innovative practice activities and prepare some prizes that female students are interested in to encourage them to join. For art students, a project to cultivate innovative technology, thinking, and ability is set up, and students are encouraged to complete these projects.

#### Strengthen the development of cultural and innovative integration industries

Under the background of comprehensively deepening the reform of the cultural system and continuously increasing the demand for cultural consumption, schools should make the integration of cultural and creative industries a reality. As a new force, cultural creativity is rising rapidly. The prosperous cultural expositions, creative design weeks, art festivals and other cultural activities have met the growing and diverse cultural needs of college students. The expansion of the cultural industry is inseparable from integrated development and innovation. College students are the main force in promoting industrial integration. Only by cultivating the innovation and entrepreneurship of college students and enhancing the cultural innovation and creative ability can we bring about the multiplier effect of “1 + 1 > 2.”

#### The creation of an innovation and entrepreneurship

The cultivation of innovation and entrepreneurship in colleges and universities requires a kind of will and cultural innovation. The process of cultivating college students’ innovation and entrepreneurship is also the process of creating a culture of innovation and entrepreneurship. The in-depth construction of innovation and entrepreneurship is an important step taken by the IEE of colleges and universities. The culture of innovation and entrepreneurship has many similarities with the excellent traditional culture of the Chinese nation, and giving full play to the advantages of the excellent traditional culture is conducive to the creation of the culture of innovation and entrepreneurship in colleges. The culture of innovation and entrepreneurship is a culture that cultivates excellent spirits. It can arouse the immeasurable enthusiasm, initiative, and sense of mission for innovation and entrepreneurship, and help the formation of college students’ innovation and entrepreneurship.

#### Provide entrepreneurial capital support for art students

From the perspective of IEE resources, the school should not only have a strong entrepreneurial campus culture but also have strong entrepreneurial education resources to provide students with sufficient entrepreneurial funds. The innovation and entrepreneurship awards are designed to issue related bonuses to drive the enthusiasm of students and provide opportunities and conditions for students to start their own businesses. School-level alumni associations can also be held. Through this model, students can help each other. Through the information, funds, contacts, and other resources provided by students with entrepreneurial experience, they can provide assistance for art students to start their own businesses.

### Measures to strengthen the innovative spirit of female art students

Innovation is a combination of new thinking and creativity. It is the source of profit for entrepreneurial enterprises and the fundamental guarantee of entrepreneurial success, and it is the creation of market value. Therefore, improving innovation ability is the primary task of entrepreneurs. Through the above research, it can be found that the innovation and entrepreneurship of male students are better than that of female students. Therefore, the innovation and entrepreneurship of female art students should be improved. The specific methods are as follows.

#### Cultivate the innovative thinking

Innovative thinking is to learn to subvert the understanding of things. Think with a skeptical mindset, whether it’s something that has been certified or something unknown. Dare to deny and transcend the confinement mode of thinking. Jumping and even absurd connections are used to prove its feasibility. These are all based on the comprehensive application of knowledge in various disciplines and are the synthesis of thinking based on certain knowledge. Therefore, it is necessary to work on insight, imagination, and intuition. Thinking is the core of intelligence.

#### Be good at summarizing experience

Innovations are not necessarily new things, and many innovations are re-creations on the original basis. By paying attention to discovering and summarizing the innovation experience of predecessors and their own failures, and according to the analysis and research of the failed experiences, new methods and approaches are summarized. Therefore, it is necessary to be good at summarizing various experiences of innovative projects, whether it is one’s own experience or that of similar projects, and choose innovative breakthroughs based on these, so that it is easier to succeed. Hence, it needs to make up for the lessons from analysis, decision-making, and foresight.

#### Be innovative in practice

Practice is the only criterion for testing truth, and it is also the only criterion for testing innovation. It is necessary to master to learn from the experience of others and the perfect combination of your own innovation, make full use of it and make it your own, and improve innovation ability and awareness in practice. Only through practice can each innovation verify its feasibility and find the value of its project. Innovations that are not proven are unreliable. Therefore, people must do their best to implement, organize and coordinate, and communicate.

#### The courage to solve the problem

Innovative activities originate from problem awareness, and the process of solving problems is the process of exercising one’s innovative ability. When encountering problems, you should pay attention to consider many aspects, and you must persevere and develop the habit of thinking. Only in this way can innovation appear unknowingly, and innovation for innovation’s sake is unlikely to occur. Only by considering and solving problems from multiple perspectives can inspiration to solve problems and innovations emerge. Problem-solving is the foundation of people’s innovative work. People can stand on the shoulders of giants to analyze, consider and solve problems. The solution to each problem requires you to exert your thinking ability and discover innovative themes and contents in the problem-solving process. Therefore, it is necessary to effort on the driving force, communication ability, and adaptability, and to be good at finding, researching, and solving problems.

### Strengthen the field of innovation in an art teaching model

#### Innovation in the teaching team

More and more scholars have begun to conduct in-depth research on the cultivation of the innovation and entrepreneurship of college students. By consulting the data, it is found that there are certain problems in the teaching team cultivated by the innovation and entrepreneurship in colleges. The following are some suggestions for the problems. The first is the quality of teachers. There are very few full-time teachers of IEE in colleges, most of them are part-time teachers, lack professional knowledge background, lack experience in cultivating the innovation and entrepreneurship of college students, and it is difficult for the teaching level to keep up with the pace of the new era. To fundamentally solve the problem of the quality of these teachers: on the one hand, professional teachers need to be introduced to inject fresh blood into the teacher team. On the other hand, there is a need for professional and normalized training for existing teachers to effectively enhance their professional level of existing teachers. The second is the number of teachers. With the continuous expansion of Chinese colleges and universities, the number of college students enrolling every year is much higher than before, but there are generally fewer teachers engaged in IEE in many universities. Teachers’ teaching is often overloaded, and it is difficult to concentrate on cultivating the innovation and entrepreneurship of college students. Due to the lack of professional teachers, it is inevitable that the content and quantity of courses must be compressed, which directly affects the quality of teaching. Therefore, it is necessary for universities to increase the number of professional teachers to ensure that the proportion of students and teachers is reasonable, scientific, and balanced. The third is the concept of teachers. Due to the constraints of traditional teaching concepts, the teaching methods of spiritual cultivation in some universities are rigid, cramming, backward and traditional. Some teachers have backward teaching concepts, and they have the wrong concept of success or failure based on scores.

#### Innovation in educational concepts

Colleges have changed the concept of cultivation. First, to understand that the key to cultivating the innovation and entrepreneurship of college students is practice. Only by carrying out practical teaching, allowing college students to fully participate in and experience the fun of innovation and entrepreneurship, can they voluntarily and actively integrate into teaching and have good teaching effects. Therefore, teachers should strengthen the practice view of teaching, integrate basic knowledge into practice, and apply and use what they have learned. Second, an excellent and advanced team of teachers. The times are constantly progressing. In the new era, teachers should give full play to their team advantages, keep learning advanced, learn from each other’s strengths and complement their weaknesses, so that their teaching concepts can keep up with the pace of the times, constantly improve their professional knowledge, and learn the advanced concepts of the elite. The formation of a professional team of teachers for the cultivation of the innovation and entrepreneurship of college students is the cornerstone of spiritual cultivation in colleges. General Secretary Jinping Xi said that the key to talent training lies in teachers. The quality of the teaching staff directly determines the ability and level of a university to run a school. “Without an excellent team of teachers, it is impossible to cultivate. Therefore, universities should continue to explore and actively establish a professional team of teachers with excellent quality, high professional level, advanced concepts, and keep pace with the times.

#### Innovation in teaching forms

The cultivation of the innovation and entrepreneurship of college students requires practice, and building a related practice platform is an effective means to create a good practice environment for spiritual cultivation, such as innovation and entrepreneurship training bases, incubation bases, and other practice platforms. First, these practical platforms help college students to broaden their horizons, apply their professional knowledge and realistic ideas to practice, and accumulate valuable experience in practice, which is conducive to the formation of innovation and entrepreneurship. The second is that these practical platforms are helpful for the personal development of college students. They will constantly temper their will in practice, have the courage to face failure, and the spirit of continuous struggle, feel the benefits of teamwork in team practice, and form a scientific adventurous spirit. In the process of innovation and entrepreneurship, the responsibility spirit of college students in the new era who dares to make breakthroughs is formed, and innovation and entrepreneurship are formed through slow and continuous accumulation and practice. Third, these practice platforms provide college students with opportunities for extensive cooperation and exchanges. Through cooperation, the success rate of college students is often much higher than that of fighting alone. Their level of innovation and entrepreneurship is improved, the field of innovation and entrepreneurship is broadened, and their advantages can be effectively brought into play.”

#### Innovation in resource allocation

While carrying out cooperation and exchanges among the government, universities, and enterprises, the proportion of resource allocation for innovation and entrepreneurship cultivation in universities should be scientifically and rationally increased. On account of the significance of the formation of the innovation and entrepreneurship of college students, it is necessary to invest more funds rationally and scientifically. Practical platforms such as innovation and entrepreneurship training bases and incubation bases all need financial support, and these practical platforms cannot be established without the necessary financial support. Seemingly, the government, universities, and enterprises have invested more money. In the long run, the achievements of innovation and entrepreneurship formed by college students through practice platforms and incubation bases and the innovation and entrepreneurship honed from them are more valuable. First, the government and universities have solved the employment problem of college students from the root and realized an efficient employment model that promotes employment through entrepreneurship. Second, the innovation and entrepreneurship achievements of college students in turn promote the innovation and development of enterprises. In general, this is a mutually beneficial investment method.

### Analysis of the implementation effect of innovation and entrepreneurship education in the modern art teaching mode

**FIGURE 4:**
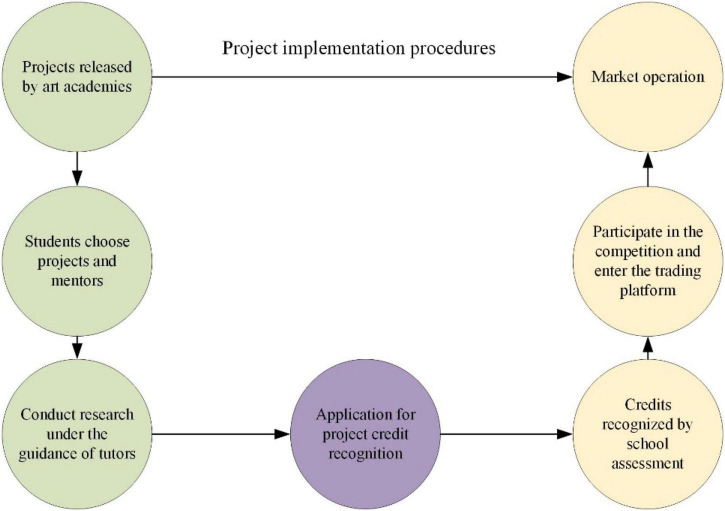
The implementation flow chart of participatory IEE in art colleges.

The basic knowledge module retains the basic content of the original professional curriculum. Its proportion has been increased to 50–60%, ensuring that knowledge in this discipline can effectively provide knowledge in other disciplines. The discipline is smoothly linked with other disciplines. The frontier knowledge unit is the frontier subject of the development and evolution of this discipline, accounting for 10–15% of the total number of students in the whole course and focusing on the coordinated development of new knowledge and market forecasting. The practical operation module is the basic knowledge of a professional course and has practical value. The market needs and adaptation module is designed for a specific market or potential need, accounting for about 10–15% of the course. It is mainly aimed at some new problems often encountered in practical applications. The professional and practical entrepreneurial integration can make full use of the school’s professional teachers and hire teachers to provide consulting services for enterprises. It can also solve the problems encountered by enterprises in entrepreneurship in time, thereby reducing the cost of enterprises (Ding and Shi, 2021). The graduation creation cycle of art colleges is long, and the task is heavy. Therefore, graduation design and creative entrepreneurship can be combined to achieve a “win-win” (Chen et al., 2019). There are two main types of research activities for students in this discipline. One is student participation, and the other is teacher participation. “Student participation” means that students choose projects according to their interests and ask teachers to guide them. “Teacher participation” refers to national, provincial, or local scientific research involving professional teachers (Alyabyeva et al., 2021). Project participatory implementation is usually a two-way choice between teachers and students. Figure 4 presents the implementation flow chart of participatory IEE in art colleges.

**FIGURE 5:**
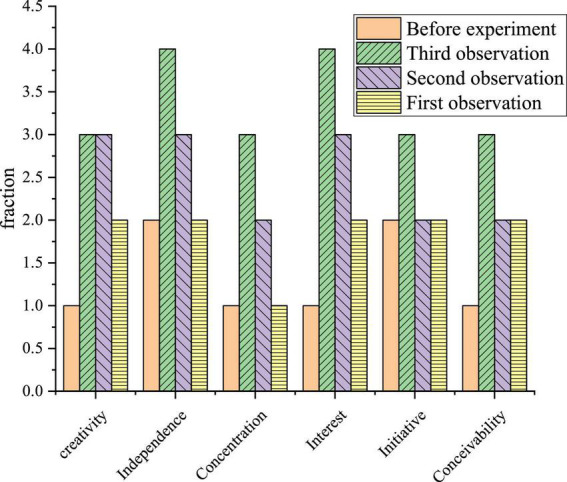
Statistics of classroom observation data of innovation and entrepreneurship teaching model.

The creativity, initiative, interest, conception, independence, and concentration of art students is expressed from six aspects. There are four options for each dimension. Each choice has a different score, which is divided into one, two, three, and four points. The highest score is four points, and the lowest score is one point. It is found that the four classroom observation trends of Xi’an Academy of Fine Arts are as follows by comparing the observation data of different classes (Figure 5).

After three rounds of activities, the researchers will assess the results of classroom observations in the three stages of the four stages of observation questionnaires. The purpose is to understand the effect of artistic activities under the IEE model. Classroom observation includes creativity, initiative, interest, ideation, independence, and concentration. Data are repeated measurements. Mauchly’s test of sphericity is adopted here. When *P* > 0.05, the analysis of variance (ANOVA) is repeated. If *P* < 0.05, Greenhouse-Geisser is used for correction. When the repeated ANOVA *P* < 0.05, the data of four-time points are compared twice, and *P* < 0.05 is divided into two groups.

Creative behavior evaluation uses repeated ANOVA. It is found that art teaching in IEE can effectively promote students’ creative play.

Evaluation of subjective initiative: Through repeated analysis of variance, *F* = 87.245, *P* = 0, indicating that the test scores at different times are statistically different, and the scores increase with the increase of time. After comparing the test situations at four different time points, it is found that there are statistical differences in the first, second and pre-experiment comparison scores (*P* < 005), indicating that under the teaching mode of innovation and entrepreneurship, art teaching can effectively improve student initiative. The test initiative scores at four different times are exhibited in Table 11.

**TABLE 11:** Comparison of initiative scores at different times.

Initiative	Mean value	Standard Deviation (SD)	*F*	*P*
The first experiment	2.05	0.557	87.245	0
The second experiment	3.12	0.650		
The third experiment	3.26	0.673		
Before experiment	1.45	0.618		

Evaluation of interest: Through repeated analysis of variance, *F* = 60.632, *P* = 0, indicating that the test scores at different time points are statistically different, and the scores increase with the increase of time. After the pairwise comparison of the test conditions at four different time points, it was found that there were statistical differences in the four observation data comparisons (*P* < 0.05). It refers that under the teaching mode of innovation and entrepreneurship, art teaching can effectively improve the interest of college students. Table 12 expresses the comparison of interest scores observed at four diverse time points.

**TABLE 12:** Comparison of interest scores at diverse times.

Interest	Mean value	SD	*F*	*P*
The first experiment	2.17	0.682	60.632	0
The second experiment	3.02	0.750		
The third experiment	3.46	0.565		
Before experiment	1.65	0.736		

Conceptual performance evaluation: The repeated variance method is used, *F* = 33.409, *P* = 0, showing that the scores at different time points are statistically different. The score increases with time. After comparing the test situations at four different time points, it is found that there is no statistical difference between the two scores of the observation before the experiment and the observation of the first teaching experiment (*P* > 005). The observed data of the second and third experiments are not statistically different (*P* > 005), but the first two (data collected before and the first experiment) and the last two (data collected in the second and third experiments) are statistically obvious (*P* < 0.05). It shows that under the teaching mode of innovation and entrepreneurship, art teaching can effectively improve the conception of college students. A comparison of the conceptual scores observed at four different time points is demonstrated in Table 13.

**TABLE 13:** Comparison of conceptual scores at distinct times.

Conceptual	Mean value	SD	*F*	*P*
The first experiment	2.03	0.727	33.409	0
The second experiment	3.01	0.828		
The third experiment	2/92	0.723		
Before experiment	1.63	0.698		

Evaluation of independence: Repeated variance method is used, *F* = 45.39, *P* = 0, indicating that the scores at different time points are statistically different, and the scores increase as time increases. After comparing the test situations at four different time points, it is found that there is no statistical difference between the before the experiment and the first experiment (*P* > 005), and the first, second and third teaching experiments have statistical differences (*P* < 0.05). It means that under the teaching mode of innovation and entrepreneurship, art teaching can effectively improve the independence of college students’ classrooms. The comparison of the observed independence scores at four different time points is shown in Table 14.

**TABLE 14:** Comparison of independence scores at different times.

Independence	Mean value	SD	*F*	*P*
The first experiment	2.09	0.765	45.39	0
The second experiment	2.85	0.755		
The third experiment	3.32	0.691		
Before experiment	1.90	0.722		

Evaluation of the learning effect of concentration: the repetition variance is 16.180, *P* = 0. It refers that the scores at different time points are statistically different, and the scores increase as time goes on. After the pairwise comparison of the test conditions at four diverse time points, it is found that there is no statistical difference in the scores before the experiment, the first experiment, and the second experiment (*P* > 0.05). The data of the second experiment and the third experiment were statistically different (*P* < 0.0). It denotes that under the teaching mode of innovation and entrepreneurship, art teaching can effectively improve the concentration of college students in the classroom. Table 15 signifies the comparison of the concentration scores observed at four different time points.

**TABLE 15:** Comparison of attentiveness scores at diverse times.

Concentration	Mean value	SD	*F*	*P*
The first experiment	2.12	0.780	16.180	0
The second experiment	2.45	0.867		
The third experiment	3.08	0.765		
Before experiment	1.98	0.950		

Through the above data analysis and three rounds of action research, it is found that the performance of art students in all dimensions has been significantly improved. Before the start of the teaching activities, the values of the various dimensions of the classroom performance of the art students are not high. When the painting activities are carried out, the art students often could not finish the activities, and some art students would give up or leave to do other things in the middle. Based on the above problems, the researchers decided to carry out art classes under the IEE model, and carry out art teaching activities in combination with the ATDE teaching model, to stimulate the interest of art students and improve teaching problems. After the second round of teaching activities, the performance values of interest, initiative, creativity, and independence show significant differences. However, due to the fact that art students have less contact with the teaching method of IEE, their emotions are not stable, and it is difficult to maintain classroom discipline, which leads to the time spent in the process being too long, and the time for other links is not enough. Their concentration is often diverted during the painting session. In the later teaching practice, the time for actual operation and work display is relatively reduced. In the “doing” link, art students are encouraged to boldly make breakthroughs and innovations in painting creation. With the development of teaching activities, they also are gradually familiar with IEE teaching resources and models, they are gradually adapting to the new teaching model, and their emotional changes are relatively stable. Therefore, in the second and third rounds of teaching actions, the numerical comparison results of the concentration and conception performance of art students denote obvious differences in succession.

The development of IEE in art colleges has its own advantages. IEE of students in art colleges must rely on its unique resource advantages. Starting from the discipline and major, taking students as the center, it will strive to explore the innovation and entrepreneurship model of college students in art colleges with strong innovation and creativity, and professional characteristics. This chapter conducts an empirical study on the IEEs of the eight major academies of fine arts, and sorts out the current situation and causes of IEEs in Chinese art academies by means of a questionnaire survey. It is believed that the main factors affecting the innovation and entrepreneurship of art students include: creativity, initiative, interest, ideation, independence and concentration. It has been verified by implementing an art education model under innovation and entrepreneurship. It has a strong guiding role and significance for the optimization and improvement of the art education model. To create an active campus cultural atmosphere of innovation and entrepreneurship, and to create the most artistic innovation and entrepreneurship incubation base, the IEE of art academies should be closely integrated with social development, and focus on the creation of a collaborative innovation mechanism.

## Conclusion

Through theoretical analysis and practical exploration, IEE is incorporated into the professional education system of art colleges. And a series of reasonable and effective IEE model groups are designed to provide a theoretical basis and guidance for the cultivation of high-quality innovative and entrepreneurial talents in art colleges. It innovatively proposes to give full play to the characteristics of art colleges, to serve local cultural inheritance and innovation and the development of cultural and creative industries, to construct an IEE model based on professional teaching, to break the traditional teaching model of separation of “foundation and creation” in art colleges, to achieve innovation and beauty, entrepreneurship and art, and the coupled development of IEE and professional education. The conclusions are as follows. First, it believes that IEE in art colleges must emphasize the integration with professional education, so that students can obtain perceptual experience through professional practice; The core content should pay more attention to integrity, cultivate the rational action of students, and use their professional advantages to seek the development space of the industry; The purpose of education should return to the person itself, highlight the subject status of students, and reflect their dignity and value; Educational methods should adopt experientially, guided and interactive teaching to comprehensively improve students’ comprehensive quality, cultivate their spirit of reflection, criticism, and innovation, and promote their comprehensive development. Second, through empirical research and interviews with students from eight art colleges, it discusses the factors that affect the education model of art colleges and creates an art teaching model under the IEE model to improve teaching efficiency. Third, through empirical analysis, the factors that have a major impact on the innovation and entrepreneurship teaching model are of great significance to the optimization and improvement of subsequent teaching methods. The outlook is as follows. First, in terms of the breadth of future research, it is necessary to carry out international cooperation, conduct the cross-border study, expand the horizons of academic exchanges, and continuously integrate new factors of the development of the times during this process. Second, in terms of the depth of research, IEE should be reflected in all aspects of higher education, and the education evaluation system should be the core content of the research. At the level of methods, systems analysis, represented by systems theory, cybernetics, and information theory, is helpful for in-depth analysis of the influencing factors of IEE. Third, because of the differences in regional culture and the unbalanced economic development, art colleges need to select reasonable dynamic indicators for observation during the implementation process to avoid the limitations of conclusions. The limitations of the study are as follows. Firstly, due to the limitation of materials on the innovation and entrepreneurship of college students and the limited level of ability, it cannot fully cover all the content, and it is insufficient. Secondly, it fails to study the changes in the learning ability of art students under the innovation and entrepreneurship teaching model in detail. It is hoped that more scholars will participate in the research to criticize and correct. Thirdly, the designed IEE model is relatively simple, and more complex and diverse factors should be included in the follow-up research.

## Data availability statement

The raw data supporting the conclusion of this article will be made available by the authors, without undue reservation.

## Ethics statement

The studies involving human participants were reviewed and approved by Malaysia City University Ethics Committee. The patients/participants provided their written informed consent to participate in this study. Written informed consent was obtained from the individual(s) for the publication of any potentially identifiable images or data included in this article.

## Author contributions

Both authors listed have made a substantial, direct, and intellectual contribution to the work, and approved it for publication.
